# Localized Merkel cell carcinoma treatment considerations: a response to the forty-year experience at the Peter MacCallum cancer centre

**DOI:** 10.1186/s12885-024-12443-y

**Published:** 2024-06-03

**Authors:** James Leigh, Kurt Gebauer

**Affiliations:** 1 University of Oxford, Oxford, United Kingdom; 2 Fremantle Dermatology, Fremantle, Australia; 3 University of Western Australia, Crawley, Australia

**Keywords:** Carcinoma, Merkel Cell, Skin Neoplasms, Australia, Radiation Oncology, Surgery, Neuroendocrine Tumour

## Abstract

Merkel cell carcinoma (MCC) is a rare but aggressive neuroendocrine tumour of the skin with poor prognosis and rising global incidence. A recently published article in BMC Cancer, titled “Merkel cell carcinoma: a forty-year experience at the Peter MacCallum Cancer Centre” (Wang et al.), provides a contemporary analysis of locoregional disease outcomes in Australia which highlights the comparative effectiveness of radiotherapy for excisions with involved margins versus wide local excision. There is a persistent lack of clear, well-defined guidelines to manage MCC in Australia despite experiencing the highest rates globally. The advanced age at onset also provides inherent challenges for optimal management and often, a case-by-case approach is necessary based on patient preferences, baseline function and fitness for surgery. This paper responds to the recently published article by Wang et al. and will expand the discourse regarding management of localized MCC. Specifically, we will discuss the surgical excision approaches; alternative treatment options for MCC including radiotherapy, Mohs micrographic surgery and novel immunotherapy agents being investigated through several clinical trials.

This journal recently published an article by Wang et al., which describes a forty-year experience at Australia’s Peter MacCallum Cancer Centre managing Merkel cell carcinoma (MCC), a highly aggressive cutaneous neuroendocrine tumor [1]. There is ongoing debate regarding management of localized MCC which we will expand upon in this article, including surgical margin considerations, immunotherapy and lessons learned from melanoma treatments.

## 1. Surgical margin considerations for localized MCC

Surgical margins are an important consideration in MCC management, particularly among elderly patients or those undergoing adjuvant radiotherapy (RT). Wang et al. conclude that “if treated with adjuvant radiotherapy, there is no difference in overall survival or disease-free survival with positive surgical margins” [1]. WLE has traditionally been recommended as a primary treatment modality with sentinel lymph node biopsy (SLNB) [2]; however, the findings by Wang et al. challenge this paradigm and aligns with the clinical outcomes which we have observed in our high-volume community dermatology clinic. Globally, guidelines are recognizing alternative first-line treatment modalities for localized MCC including RT, Mohs micrographic surgery and immunotherapy.

The National Comprehensive Cancer Network (NCCN) has developed revised guidelines for the management of MCC [3]. Wang et al. reference the 2021 NCCN guidelines, which recommend 1–2 cm margins as definitive treatment for localized MCC in low-risk cases with absent risk factors (larger primary tumor (> 1 cm); chronic T-cell immunosuppression, HIV, chronic lymphocytic leukemia (CLL), solid organ transplant; head/neck primary site; lymphovascular invasion (LVI) present) [3]. However, an updated version of the NCCN guidelines now exists and instead recommends that “surgical margins should be balanced with morbidity of surgery, with surgical goal of primary tissue closure to avoid undue delay to adjuvant RT” [4]. Their updated terminology signals a preference for adjuvant RT over clear surgical margins, in the event of delayed RT due to wound healing for WLE. These guidelines also include Mohs micrographic surgery as an option for primary excision, which has recently been recognized as a comparable treatment for localized MCC [5] but was not acknowledged in Wang et al.’s article. Further research is needed to identify the most effective excision technique which considers the importance of time-sensitive adjuvant radiotherapy, particularly in comorbid or surgically unfit patients more common among the elderly.

## 2. The role of immunotherapy in localized MCC

Immunotherapy has significantly changed the landscape of systemic treatments for metastatic cancer. Its role in localized cutaneous malignancy is also being increasingly recognized, particularly among melanoma and MCC. Since the publication of the Peter MacCallum forty-year experience managing MCC [1], the NCCN has updated their guidelines to include immunotherapy for locally recurrent N0 (local) disease if surgery and radiotherapy are not viable treatment options [4]. The specific immunotherapies include pembrolizumab and retifanlimab-dlwr which are approved for use in America, while avelumab is the approved agent in Australia. Of note, avelumab is only indicated in metastatic disease (stage IV) in Australia at the time of writing; however, the I-MAT study is investigating the efficacy of avelumab for stage I-III MCC [2]. Existing immunotherapy trials include the ADMEC-O trial (NCT02196961) which is investigating the efficacy of adjuvant nivolumab monotherapy in patients with completely resected MCC [6]. Other trials include America’s ADAM trial which investigates the efficacy of avelumab for regional disease that has spread to the lymph nodes [7], and the STAMP clinical trial which investigates the efficacy of adjuvant pembrolizumab after surgery for stage I-III disease [8].

Adjuvant immunotherapy presents a viable alternative treatment in patients who are unfit for major surgery and with logistical barriers to RT, often delivered over 20–30 separate sessions. In our experience, a sizeable cohort of MCC patients are considered unfit for major surgery due to their advanced age at diagnosis, poor mobility and comorbidities. Furthermore, several of our patients declined RT due to difficulty with transportation particularly among elderly patients with mobility difficulties. Although immunotherapy can be very costly and cause unwanted side effects, it may be an appropriate treatment option in patients with MCC who are not amenable to WLE or RT, given the rarity of this cancer and demonstrated benefit in systemic disease. Effective patient selection for immunotherapy is important to maximize benefit considering both high costs and toxicity profiles associated with treatment. This is evident in countries such as Norway where immunotherapy is not considered cost-effective [9]. Furthermore, immunotherapy may provide benefit in non-surgical patients where sentinel lymph node biopsy is not possible.

## 3. Lessons learned from melanoma

Melanoma treatments have rapidly advanced in the past decade, and several findings may be translatable to the management of MCC from a surgical and medical perspective. Immunotherapy provides significant patient benefits, including improved disease-free survival and overall survival. Given the high metastatic potential among patients with invasive melanoma, patients are now being considered for immunotherapy even in localized disease. Indeed, a recent study by Eggermont et al. (KEYNOTE-716) demonstrated the benefits of adjuvant pembrolizumab for preventing disease recurrence or death in stage IIa and IIb melanoma after excision [10]. As we have described, there is comparable MCC research relating to adjuvant immunotherapy through the STAMP trial which is due for completion in 2025 [8].

On a surgical front, the MelMarT-II trial is investigating the clinical outcomes of 1 cm vs. 2 cm margins for patients with localized melanoma [11]. Wide local excision has long been considered the gold-standard surgical treatment for melanoma and Merkel cell carcinoma; however, this surgical approach is being reconsidered to minimize unnecessarily wide margins and associated complications. Conducting an equivalent randomized controlled trial for the surgical management of MCC presents a challenge due to its relative rarity. However, the outcomes of MelMarT-II should be contextualized to MCC including the cost-effectiveness of WLE and patient quality of life implications after surgery.

## Conclusion

Merkel cell carcinoma is a highly aggressive cutaneous neuroendocrine tumor. Although rare, it most commonly affects elderly patients, many of whom have comorbidities that may limit treatment options including WLE or RT. Wang et al. provide an important and contemporary description of their forty-year experience in managing MCC in Australia, a country with the highest incidence of MCC globally. WLE may not be the best treatment option for all patients, and clinicians should be aware of the various treatment options which exist for localized MCC. These include WLE and sentinel lymph node biopsy with or without adjuvant RT; Mohs surgery; isolated RT; and more recently, immunotherapy. Less invasive treatments for MCC do exist and may be favorable among patients with poor functional status or contraindications to surgery. Further prospective research which acknowledges the clinical challenges of advanced age at diagnosis, for example a randomized controlled trial, may augment the evidence for the management of localized MCC among elderly patients.

## Data Availability

Not Applicable.
